# Criminal governance and extortion in informal economies: fear as a mechanism of territorial control in Lima

**DOI:** 10.3389/fsoc.2026.1795425

**Published:** 2026-05-12

**Authors:** Javier Hildebrando Espinoza Escobar, Samantha Sotelo-Llancari, Viviana Isabel La Rosa Salvador

**Affiliations:** 1 Universidad Autónoma del Perú, Lima, Peru; 2 Pontificia Universidad Católica del Perú, Lima, Peru; 3 Universidad Privada Norbert Wiener, Lima, Peru

**Keywords:** criminal governance, economic informality, extortion, affective regimes of fear, urban marginality, digitalized coercion, Global South

## Abstract

**Introduction:**

This article examines extortion as a form of criminal governance embedded in contexts of high economic informality, focusing on the case of San Juan de Lurigancho, Lima. The study conceptualizes extortion not merely as a criminal act, but as a socioeconomic mechanism that regulates territory, economic activity, and everyday life in the absence of effective state protection.

**Methods:**

Drawing on a phenomenological qualitative research design, the study involved 20 in-depth interviews with economic agents with constrained agency, community leaders, and judicial actors to explore the dynamics of extortion and its impact on social and economic regulation.

**Results:**

The findings show that extortion operates as a system of informal taxation sustained through the strategic production and normalization of affective regimes of fear, which function as a technology of social control. This regime is increasingly mediated by digital platforms, enabling the viralization of threats and a digitalization of coercion. Consequently, violence becomes routinized, social trust erodes, and silence is institutionalized as a survival strategy. Economic agents deploy adaptive responses ranging from pragmatic negotiation to fragile forms of collective organization.

**Discussion:**

The article expands the literature on criminal governance by demonstrating how economic informality, selective state presence, and digitally mediated coercion converge to produce a hybrid system of regulation. This study contributes to broader debates on informality, violence, and governance in urban contexts of the Global South.

## 1 Introduction

Extortion has become a mechanism of informal governance and territorial control linked to the expansion of the informal economy in marginalized urban contexts of the Global South. As reported by the National Institute of Statistics and Informatics (2025), in Peru approximately 70.7% of the economically active population works outside the legal framework. In this way, informality has given rise to an economy that operates according to its own rules, parallel to state regulation. This situation generates labor precarity, low incomes, and limited institutional presence, which in turn fosters the emergence of criminal networks and mafias that impose order and authority through violence or the threat of violence.

In this context, extortion functions as a form of “informal taxation” through which criminal governance actors regulate economic activities, collect fees, and provide a false sense of security. This phenomenon constitutes a form of criminal governance in which violence substitutes for the state, establishing dynamics of social and territorial control. At the same time, the informal economy not only facilitates this process by developing in areas with weak state oversight, but is also reinforced by extortion itself, generating a vicious cycle that perpetuates marginalization and weakens the social fabric.

The relationship between informality and extortion highlights how the absence of inclusive policies and sustained formal employment allows criminal structures to consolidate as alternative forms of power in the urban peripheries of the Global South. However, although the literature on organized crime and the informal economy has tended to address these phenomena separately, significant gaps remain regarding the everyday experiences and coping strategies of economic agents who operate at the intersection of both worlds. In this regard, the study by Lazo and Rivas (2022) provides valuable evidence by showing that participants not only endure economic coercion, but also negotiate, resist, and reinterpret threats according to cultural frameworks shared with their aggressors. Through a micro-level analysis of telephone interactions, a complex communicative dynamic is revealed in which violence, courtesy, and familiarity intertwine to sustain asymmetric yet negotiated relationships. Thus, extortion emerges not only as an economic mechanism of control, but also as a socially and culturally situated practice that reflects the structural fragility of those participating in the informal economy.

In the Peruvian case, the district of San Juan de Lurigancho constitutes a critical setting for examining the interrelationship between economic informality, social vulnerability, and criminal governance networks. Its high population density and marked dependence on informal employment reflect the structural conditions that, according to Kamichi (2023), define the country’s informal economy: low incomes, limited productivity, and weak state presence. In this context, informality becomes not only a survival strategy, but also fertile ground for the consolidation of forms of criminal governance that replace state functions. Extortion networks take advantage of precarity and institutional distrust to impose mechanisms of economic and social control, normalizing violence as part of everyday life.

In San Juan de Lurigancho, these criminal groups operate through a system of illegal charges or “security fees” primarily targeting merchants, transport workers, and informal workers, who must pay under threat as a form of protection. This mode of operation is sustained by state absence and the naturalization of violence in spaces dominated by the informal economy. For many affected individuals, extortion becomes a routine practice that must be negotiated or accepted as part of the cost of working, giving rise to various strategies of adaptation and resistance ranging from silence to collective organization. In this sense, extortion in San Juan de Lurigancho transcends the purely criminal and is configured as a mechanism of economic and social regulation that reflects the structural tensions of contemporary urban Peru.

Despite the growing body of literature on organized crime in Latin America, most studies have traditionally focused on the macro-logistics of drug trafficking or the formal security responses of the State. Significant gaps remain regarding the everyday micro-dynamics of extortion within high-density informal economies. While authors like Kamichi (2023) and Lavado (2024) have provided critical insights into the structural precarity and the general index of citizen insecurity in Peru, there is a lack of qualitative evidence on how these conditions facilitate hybrid systems of criminal governance. Furthermore, although Ginocchio (2022) has examined ‘legalized extortion’ by state actors in hubs like Gamarra, and Lazo and Rivas (2022) have analyzed communicative patterns in threats, the current literature has yet to fully address how digital platforms reconfigure territorial control through the digitalization of coercion and how fear is deployed as a deliberate technology of social regulation. Taken together, this article seeks to analyze the relationship between informality and extortion from a socioeconomic and cultural perspective, exploring how structural precarity and state absence contribute to the consolidation of forms of informal power. The study focuses on understanding the experiences, perceptions, and strategies of economic agents in contexts where violence is normalized as part of the everyday economy.

## 2 Literature review

Economic informality generates and perpetuates structural vulnerability in low-income communities through interconnected mechanisms. Informal insurance arrangements, such as savings, asset sales, and reciprocal exchanges, provide limited and insufficient coverage against localized risks (Miao, 2016; Morduch, 1999).

The relationship between economic vulnerability and informality operates as a feedback loop: greater vulnerability deepens dependence on informal practices (Çarkoğlu and Eder, 2006, unpublished manuscript). Within this feedback loop, gender operates as a key structuring mechanism, as many women in informal economies face restricted economic options and rely on low-yield informal credit. At the same time, informal arrangements in housing, services, and employment sustain short-term survival while simultaneously consolidating marginality (Kolling, 2022). In this way, informal workers are pushed toward increasingly informal solutions, reproducing structural disadvantage (Çarkoğlu and Eder, 2006).

Consequently, criminal violence becomes inseparable from economic vulnerability: in marginalized territories, not only do material precarities persist, but coercive mechanisms also emerge that further deepen structural fragility. In contexts characterized by high informality and weak or selective state presence, extortion emerges as a mechanism that transforms economic vulnerability into systematic coercion, allowing both criminal and parastatal actors to extract resources while exercising territorial and economic control.

Research on extortion networks in marginalized urban environments reveals complex dynamics of territorial control and economic coercion. In El Salvador, gangs create “fragile assets” through the exercise of sovereign power, rendering people and property vulnerable to extortion, a process facilitated by state taxation and the abandonment of marginalized territories (Neu, 2023). Small businesses respond strategically to these practices through a range of actions that include evasion, adaptation, negotiation, and resistance, depending on the resources at their disposal. In Nigeria, drug traffickers negotiate police extortion through temporary agreements, reflecting active forms of resistance (Nelson, 2024). Together, these cases illustrate how extortion operates through diverse coercive arrangements in contexts where state authority is fragmented, informalized, or selectively enforced.

In Colombia, merchants develop everyday coping mechanisms in response to extortion within a context in which organized crime operates as a “parasite” that disrupts and distorts the material practices necessary for the reproduction of economic life (Fernández, 2022). Along similar lines, Miller and Rettberg (2024), in a study of small and medium-sized enterprises in Medellín, argue that extortion has become normalized as a structural component of economic activity. They identify three predominant strategies adopted by merchants: compliance, where extortion is assumed as a cost of doing business; avoidance, through mobility or maintaining a low economic profile; and mitigation, via limited collective actions, such as the hiring of private security services. As their study demonstrates, violence not only extracts economic resources but also reconfigures business practices, social relations, and expectations of survival, without generating real incentives to challenge the existing criminal order.

In the Peruvian case, Ginocchio (2022) provides a crucial nuance by demonstrating that extortion is not exercised solely by criminal organizations, but also by state actors through formally institutionalized mechanisms. His study of the commercial hub shows how local authorities implement coercive charging regimes, such as arbitrary fines, payments for tolerance, and discretionary permits, that function as a form of “legalized extortion,” particularly targeting informal merchants. Although these mechanisms are formally embedded within administrative frameworks, they reproduce the logic of violent extraction characteristic of organized crime, thereby reinforcing economic vulnerability and merchants’ dependence on parastatal power networks. The case of Gamarra illustrates that, in contexts of high informality, the boundary between state regulation and extortionary coercion becomes blurred, expanding the range of actors embedded in the economic dynamics of precarious territories.

Such practices cannot be understood as isolated anomalies, but rather as part of a broader structural logic underpinning the functioning of the penal system. From the perspective of critical criminology, Baratta (2004) argues that the penal system fulfills a central role in the preservation and reproduction of social structure by operating selectively upon subordinate sectors. In this sense, criminal law does not primarily function as a mechanism of universal protection, but rather as a device of social control that concentrates punitive intervention in contexts of economic marginality.

This penal selectivity contributes to reinforcing the structural vulnerability of socially disadvantaged groups, who experience more intense forms of state control without equivalent access to effective mechanisms of protection. From this analytical framework, it becomes possible to understand why, in scenarios of high economic informality, small merchants are simultaneously exposed to criminal coercion and to forms of state regulation that, far from guaranteeing rights, reproduce relations of subordination and dependence.

Kamichi (2023) highlights that informality is strongly associated with poverty and low productivity. Informal workers earn incomes three times lower than those of formal workers and are concentrated in subsistence microenterprises. This precarity not only reproduces economic vulnerability, but also creates conditions conducive to criminal coercion, in which extortion becomes embedded as a mechanism of violent extraction. From a political economy perspective, theoretical models similarly show that large informal sectors generate incentives for coercive forms of regulation and protection in contexts of weak legal enforcement (Sarkar and Sinha, 2022).

State responses to criminality in urban contexts marked by informality are often implemented through strategies of public space control inspired by situational crime prevention and theories such as “broken windows” or “zero tolerance.” As García (2012) notes, these approaches prioritize the reduction of criminal opportunities and the maintenance of order through mechanisms of surveillance and control, while leaving the structural causes of crime largely unaddressed. In contexts of inequality, such strategies tend to concentrate state intervention on marginalized populations and informal activities, reinforcing coercive regulatory practices that deepen social vulnerability rather than reduce it, and redefining state presence primarily in terms of control and discipline over public space.

Such interventions do not eliminate criminality; rather, they reconfigure the terrain upon which other actors exercise regulatory and control functions. Albarracín (2023) explains that organized crime in Latin America operates less as a purely criminal structure and more as a form of governance, capable of imposing rules, disciplining territories, and contesting the state’s capacity for social regulation. From this perspective, extortion is not merely an economic mechanism for resource extraction, but also a form of territorial control that reproduces local hierarchies and consolidates the presence of criminal organizations in contexts where state institutional capacity is weak or selectively applied.

Similarly, Díaz (2023) demonstrates that in impoverished and highly informal territories, where state presence is intermittent or selectively enforced, forms of a “parallel state” emerge, sustained by criminal organizations that regulate everyday life, impose norms, administer conflicts, and manage coercive mechanisms of violent taxation. In such contexts, criminal governance consolidates control through violence and intimidation, effectively substituting basic state functions.

As Kamichi (2023) argues, Peruvian informality operates as a parallel economy with its own rules, characterized by low incomes, low productivity, and the absence of legal benefits. Because informality constitutes a structural condition, policies aimed at reducing labor costs or simplifying bureaucratic procedures have had minimal effects. This informal structure, accounting for over 70% of national employment, shapes territories where the state’s presence is weak, creating favorable conditions for the spread of extortion networks.

In the Peruvian case, extortion takes on a particularly critical expression in San Juan de Lurigancho, a district characterized by high levels of victimization and perceived insecurity, within a context of weak institutional control (Valdés and Basombrío, 2025). High population density, widespread informal commerce, and limited institutional enforcement capacity configure an environment conducive to extortion networks. This scenario positions San Juan de Lurigancho as a key case for understanding how economic informality and organized violence intertwine, reproducing structural vulnerabilities and eroding community cohesion (Lavado, 2024). This case illustrates broader patterns identified by Kamichi (2023), whereby informality is concentrated in impoverished territories with weak institutional presence, creating structural conditions that facilitate the emergence of parallel regulatory mechanisms such as extortion.

From a political economy perspective, extortion can be understood as a structural component of informal economies rather than as an external criminal distortion. Formal economic models show that informal production often depends on coercive intermediation, whereby extortion operates as a facilitating mechanism that enables informal firms to survive in contexts of weak legal protection and regulatory exclusion (Mandal et al., 2014). In this framework, economic reforms may unintentionally expand extortionary activities by increasing the size of the informal sector.

Taken together, these studies suggest that extortion functions through the deliberate production of vulnerability, while those targeted develop nuanced strategies of negotiation and resistance. From this perspective, extortion not only reproduces structural vulnerability but also transforms social relations through fear as an organizing principle of everyday life.

Within this same framework of structural vulnerability, violence and coercion intersect with economic informality, shaping contexts in which extortion emerges as a parallel mechanism of control and domination. In this way, fear plays a significant role in shaping social interactions and moral frameworks in urban communities with high levels of criminality through multiple interconnected mechanisms. In high-crime contexts, fear generates ambiguous social climates that reconfigure and destabilize relationships between residents and authorities, while simultaneously producing competing moral discourses regarding the legitimacy of violence (Warburg and Jensen, 2020).

Building on this focus on fear and perceived vulnerability, studies such as those by Vallejo-Robalino et al. (2024) illustrate how these dynamics materialize in everyday urban space. Drawing on social cartographies in working-class neighborhoods of Guayaquil (Ecuador), the authors demonstrate how insecurity and organized violence generate processes of symbolic and material boundary-making that reconfigure social practices and neighborhood experience. Their findings show that, in contexts of high informality and weak state presence, fear becomes internalized and normalized as a principle organizing space, giving rise to strategies of self-protection, isolation, and community-based control that do not reduce violence but rather manage it. This approach is particularly useful for understanding extortion not only as a mechanism of economic extraction, but also as a form of social regulation and informal governance in precarious urban territories.

Fear has a distinct impact on women’s spatial behavior. Among those who feel most insecure, the restriction of social ties within the neighborhood, rather than fear of crime itself, drives the avoidance of certain places (Yates and Ceccato, 2020). Similarly, young people in dangerous neighborhoods develop sophisticated strategies for managing space and navigating their environments, including avoidance, hypervigilance, self-defense, and emotional regulation, with significant variations by gender (DaViera et al., 2020). More broadly, levels of community fear are shapen by demographic factors such as gender, age, race, and socioeconomic status, and are associated with perceptions of social disorganization and lower social cohesion (Helfgott et al., 2020). These findings show that fear fundamentally reshapes social relations, moral reasoning, and spatial practices in urban communities exposed to high levels of criminality.

From this cognitive perspective, Bernhard and Cushman (2022) show that extortion is sustained through the activation of intuitive threat responses, combining coercion and promises to induce cooperation in deeply unequal exchanges. Under these conditions, economic agents tend to accept unfair agreements as a strategy to avoid greater losses, even when they recognize their exploitative nature. This phenomenon, which the authors term “the dark side of reciprocity,” helps explain how extortion can become normalized in everyday life and persist as a stable arrangement, sustained by fear rather than legitimacy, thereby shaping economic decisions and moral judgments in contexts of high vulnerability.

Mamani-Benito et al. (2025) develop and validate a brief scale to assess concern about becoming a victim of extortion among Peruvian citizens, providing an empirical operationalization of fear and perceived vulnerability in contexts of criminal coercion. In their theoretical framing, this concern is described as involving cognitive, emotional, and behavioral elements, including avoidance and digital self-censorship; however, their empirical analyses support a brief unidimensional measure. The study highlights the relevance of quantifying the emotional impact and perceived vulnerability associated with extortion in contexts where weak state control and informality are present.

Moreover, recent studies on social perceptions of organized crime reinforce the idea that extortion produces broader emotional climates of fear and insecurity that reconfigure everyday life. Alvarado et al. (2025) show, through big data analysis and text mining of more than 11,000 posts on the X platform, that terms such as extortion, protection rackets, violence, business closures, and corruption occupy a central place in public discourse. The high frequency of these mentions serves as an indicator of how criminality affects not only local economies but also collective perceptions of risk and social cohesion. Additionally, their emotional analysis reveals the predominance of fear, anger, and sadness, demonstrating that extortion operates not only as a coercive economic mechanism but also as a producer of an emotional climate that shapes community behavior and self-protection strategies.

Findings in the recent literature confirm that these dynamics of fear and cooperation not only structure community life but also shape the everyday experiences of informal micro-businesses. Alvarado (2025) shows that small merchants understand exclusion as a permanent threat that shapes basic economic decisions, including opening hours, stall location, investment in improvements, and even permanent closure, while also generating an emotional climate marked by fear, helplessness, and anger. Qualitative analysis reveals that many micro-entrepreneurs ultimately come to normalize extortion as “just another cost of doing business,” while others develop strategies of avoidance, negotiation, or periodic payment as mechanisms of survival. In this way, extortion operates not only as a mechanism of economic extraction but also as a phenomenon that reorganizes everyday life, social relations, and self-protection practices in contexts of high informality.

In contexts marked by fear and coercion, forms of resilience sustained by social capital and community networks emerge. Social capital facilitates community resilience through learning, collective action, moral or civic responsibility, and the circulation of information (Zhao et al., 2025). Networks can transform individual fear into collective anger and provide forms of shielding against coercion (Ley, 2022). In the context of extortion, collective efficacy, understood as neighborhood knowledge and participation, increases the likelihood of reporting. (Dulin, 2025). According to Wilkin et al. (2019), social network analysis and bottom-up mapping of local relationships are critical methodological tools that enable the design and planning of more sensitive and effective policies to strengthen community resilience, while avoiding the failures associated with top-down implementations. Trust building, information exchange, and the capacity for collective action within social networks emerge as central components of community resilience in the face of criminal threats.

Along these lines, economic informality, extortion, and fear constitute an interconnected web that reproduces structural vulnerability and limits communities’ capacity to confront criminal threats. At the same time, social networks and social capital demonstrate significant potential for fostering resilience, although this potential is conditioned by inequalities related to gender, resources, and community cohesion. In this context, it becomes necessary to undertake research that analyzes the dynamics of extortion in settings of high economic informality, examining its operational mechanisms, its impact on social vulnerability, and the structural barriers to accessing justice, with the aim of proposing comprehensive intervention guidelines.

## 3 Methods and corpus of analysis

### 3.1 Research setting and participant context

The study was conducted in San Juan de Lurigancho (SJL), the most populous district in Peru, with a population exceeding 1.2 million. According to official data (INEI, 2025), SJL represents a critical socioeconomic landscape where high population density coincides with a national informal employment rate of 70.7%. This urban environment is characterized by rapid, unplanned expansion and intermittent institutional presence, configuring what Lavado (2024) defines as a territory of high vulnerability to criminal governance. In alignment with INEI (2025) reports indicating that 72.8% of the Peruvian workforce is concentrated in micro-enterprises (1 to 10 workers), the participants in this study consist of micro-entrepreneurs from highly exposed informal sectors: street vendors, small retail shop owners (bodegueros), and informal transport operators (mototaxistas). These subjects operate in a context of structural precarity where the cupo (extortion fee) is perceived as a routine ‘informal tax’ necessary to maintain access to their workspace.

The study is situated within the phenomenological interpretive paradigm, aimed at capturing the lived essences of extortion in contexts of economic informality in San Juan de Lurigancho, Lima, Peru. A descriptive–exploratory qualitative research design was adopted, non-experimental and cross-sectional in nature, integrating ethnographic fieldwork and in-depth semi-structured interviews in order to triangulate subjective meanings without variable manipulation.

Regarding participants, a total of 20 individuals were selected through purposive and snowball sampling, distributed across three groups. The inclusion of judicial actors responded to the need to contrast experiences of criminal coercion with institutional logics of state control and regulation, in line with contributions from critical criminology concerning penal selectivity and coercive regulation in contexts of economic informality. The groups were organized as follows: (1) eight direct participants (informal merchants and transport workers with recent experience of direct or indirect extortion); (2) six community leaders; and (3) six judicial actors (prosecutors and police officers). Inclusion criteria comprised being over 18 years of age, direct exposure to extortion within the previous 24 months, residence or employment in informal areas of San Juan de Lurigancho, and provision of informed consent. Exclusion criteria included acute psychological vulnerability or links to criminal activity. The first six interviews were conducted as a pilot phase.

Initial contact was established through community organizations and local institutions, followed by interviews conducted in safe and neutral locations (average duration: 58 min), which were audio-recorded and transcribed verbatim with minor corrections. Ethnographic observation encompassed 48 h across varied time periods. This phase enabled the documentation of spatial avoidance practices, self-protection routines, and everyday forms of fear management associated with territorial control, dimensions central to the theoretical framework linking economic informality, coercion, and structural vulnerability. Post-collection procedures included anonymization, systematization into triangulated matrices, and secure data storage. The pilot phase allowed for refinement of the interview guide prior to conducting the 14 main interviews.

About ethical considerations, the study adhered to the Belmont principles (autonomy, beneficence, and justice).

The Research Ethics and Scientific Integrity Committee of the Universidad Autónoma del Perú reviewed the study documentation and determined that the research did not require prior formal ethical approval, as it constituted an observational and non-interventional academic study. The Committee granted a waiver of written informed consent due to the highly sensitive nature of the topic and the potential risks associated with written documentation. Verbal informed consent was obtained from all participants prior to data collection.

It included verbal informed consent, participant pseudonymization, an emotional support protocol, post-analysis data destruction, and a safety plan (participant accompaniment and emergency communication procedures).

### 3.2 Data analysis

Data analysis was conducted using ATLAS.ti version 9 through a directed thematic analysis. The process began with an intensive open coding phase, during which 1,872 codified segments (quotations) were identified across the total corpus of 20 interviews. These initial segments were then systematically synthesized through axial coding to identify recurrent patterns and eliminate conceptual redundancies, eventually consolidating the final system of 28 subcategories. Theoretical saturation was determined through a constant comparative method; by the 16th and 17th interviews, the research team observed that new data provided redundant information regarding the core categories of criminal governance and normalized fear. The final three interviews (18–20) were used to confirm that no new conceptual properties were emerging, ensuring interpretive rigor through intra- and inter-group triangulation and memo auditing. The directed thematic analysis was grounded in sensitizing categories derived from the theoretical framework (structural vulnerability, economic informality, coercion, fear, normalization of extortion, and criminal governance) which guided the initial coding process without constraining the emergence of inductive subcategories. This strategy enabled an explicit articulation between theoretical concepts and the empirical meanings produced by participants, strengthening analytical coherence between the conceptual framework and the data interpretation process.

### 3.3 Validation

Content validity of the interview guide was established through qualitative expert judgment, aimed at assessing its theoretical coherence and phenomenological adequacy for capturing lived experiences of extortion in contexts of informality. Five experts in interpretive criminology, criminal governance, and phenomenological analysis reviewed the initial instrument based on criteria of theoretical relevance, alignment with the study objectives, conceptual clarity, and the evocative capacity of the questions.

The process prioritized a reflexive evaluation of the instrument’s ability to elicit dense and meaningful narratives, rather than mere formal adequacy of individual items. As a complementary quantitative support, a four-point Likert scale was used to systematize expert judgments, yielding an aggregated Aiken’s V of 0.89 (95% CI: 0.82–0.95), indicating a high level of agreement regarding the instrument’s construct representativeness. Based on qualitative feedback, four questions were reformulated to reduce semantic ambiguities and eliminate local jargon,two conceptually redundant items were merged, and the sequence of the guide was reorganized to promote a narrative flow consistent with the phenomenological logic of the interview.

Empirical validation of the guide was conducted through a qualitative pilot using the first six interviews from the total corpus, selected for their diversity of profiles and experiences. Transcripts were analyzed in ATLAS.ti version 9 using initial open coding, co-occurrence analysis, and thematic density exploration, with the aim of assessing the analytical productivity of each question and its contribution to the study’s theoretical categories. Pilot analysis allowed for the identification of questions with low analytical yield, as well as thematic redundancies among items activating similar meaning clusters. Additionally, early stabilization of the main narrative axes associated with the modus operandi of extortion and the normalization of fear was observed, indicating preliminary saturation of these cores. These findings led to the elimination of low-yield items, the merging of redundant questions, and the incorporation of exploratory probes designed to deepen emerging dimensions. The refined guide was subsequently applied in the remaining fourteen interviews, optimizing analytical efficiency and the semantic density of the final corpus.

### 3.4 Analytical reliability

Analytical reliability was strengthened through an intercoding protocol aimed at enhancing the interpretive coherence of the categorical system. Two researchers independently coded a randomly selected subset representing 20% of the corpus, using a previously agreed hierarchical category scheme. Discrepancies were examined through iterative sessions of reflexive discussion, supported by comparative visualization of codes and segments in ATLAS.ti. This process enabled the refinement of operational definitions and the consolidation of shared interpretive criteria. As complementary quantitative support for the level of convergence achieved, an adapted Kappa coefficient for qualitative data was calculated (*κ* = 0.81; 95% CI: 0.74–0.88), indicating substantial agreement. The final categorical system, consisting of three primary categories and twenty-eight emergent subcategories, was consolidated through intra and intergroup triangulation, as well as external auditing by a third coder. The analytical process was fully documented through memos and audit records, ensuring traceability throughout all stages of analysis.

### 3.5 Epistemological justification

The exclusion of traditional psychometric procedures, such as Cronbach’s alpha, reflects their ontological inadequacy within interpretive qualitative paradigms, which are fundamentally incompatible with assumptions of unidimensionality and metric equivalence. Instead, these paradigms prioritize theoretical saturation, reflexivity, and the shared construction of meaning. Cronbach’s alpha presupposes numerical intervals and nomothetic generalization, which risks distorting subjectively lived phenomena such as experiences of extortion, where analytical reliability emerges from semantic density and interpretive consensus rather than from aggregated internal consistency. When applied to heterogeneous qualitative categories, such indices may produce artificially inflated estimates that obscure, rather than clarify, meaning.

This methodological choice aligns with qualitative guidelines, including COREQ, SRQR, and TRUST, which emphasize procedural rigor through transparent coding protocols, reflexive analytic practices, intercoder convergence, and multimodal triangulation, rather than reliance on positivist reliability metrics. Within this framework, the adopted methodological design enables a coherent operationalization of the concepts developed in the theoretical framework, capturing extortion as a lived and situated experience in contexts of high economic informality, where criminal coercion, selective state regulation, and everyday survival strategies intersect.

## 4 Results

**Figure 1:**
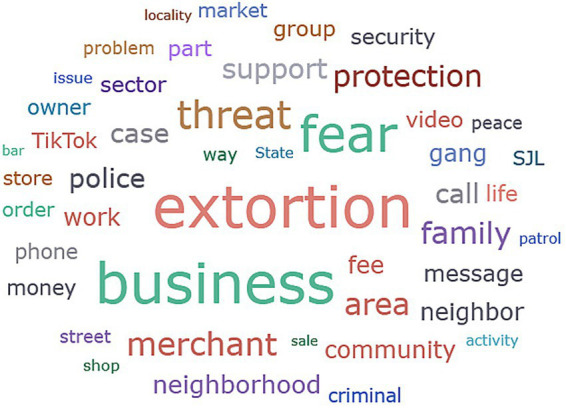
Tree Map of conceptual frequency and thematic clusters. Source: analysis in ATLAS.ti based on 20 in-depth interviews. The qualitative frequency analysis, visualized in the figure, reinforces the prevalence of extortion as the central phenomenon (*n =* 50), inextricably linked to the emotional climate of fear (*n =* 40) and the normalization of ‘protection fees’ (*n =* 9). Notably, the emergence of digital terms such as ‘WhatsApp’ and ‘TikTok’ within the top thematic clusters provides empirical evidence for the digitalization of coercion.

**Figure 2:**
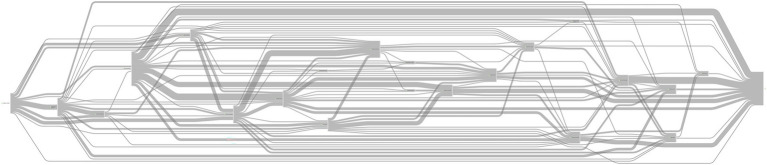
Sankey diagram of code co/occurrence: from structural state absence to socio-emotional impacts. Source: prepared by the authors using ATLAS.ti 9.

The phenomenological analysis of the narratives collected in San Juan de Lurigancho reveals that extortion operates as the central axis of a criminal ecosystem of informal governance, a complex system in which economic, social, emotional, and digital dimensions intertwine to produce and reproduce vulnerability. The findings, visualized in the Sankey diagram of code co-occurrence (Figure 1) and synthesized in the word cloud (Figure 2), are not presented as isolated themes but as nodes within a densely interconnected network. The Sankey diagram, in particular, functions as a map of causal and coexistent relationships, showing how robust flows link state absence with criminal governance, normalized fear, and a culture of silence, thereby configuring a parallel circuit of power. This section unfolds this architecture through four interrelated pillars.

The Sankey diagram (Figure 2) provides a sophisticated visualization of the co-occurrence network, illustrating the flow from structural causes to socio-emotional consequences. The model confirms the causal hierarchy where state absence and institutional distrust act as the primary foundational nodes. This flow demonstrates how state intermittency enables criminal actors to impose a coercive goivernance system through a culture of silence an fear learning, resulting in a pervasive sense of vulnerability.

### 4.1 The production of an emotional regime: fear as a technology of internalized social control

The first salient finding is the transformation of fear from a reactive emotion into an internalized and normalized technology of social control. The word cloud, in which “fear” and “terror” emerge prominently alongside “tranquility” (as an absent or longed-for signifier), provides a lexical snapshot of this affective landscape. Participants’ accounts go beyond describing isolated incidents; they detail a permanent reconfiguration of subjectivity and spatial experience. As one merchant explains: “You no longer feel safe, not even inside your own shop.” “When it gets dark, I already start thinking about closing quickly” (E2). This alteration of habits and perceptions of safety in everyday spaces reflects the previously discussed phenomenon of fear-induced spatial avoidance.

The analysis identifies two key mechanisms underlying this process of production:

1. Digital viralization of terror: Platforms such as TikTok and WhatsApp (key terms in the word cloud) have become instruments for the mass and performative dissemination of threats. A delivery worker describes the process: “They upload videos on TikTok showing businesses in the area, family names, or addresses, hinting that they are ‘marked.’ And just that is enough to scare people” (E4). This digital “marking” functions as a form of public shaming and collective warning. Threats are not issued privately; instead, a spectacle of power is staged for a broad audience, exponentially amplifying its psychological impact and disciplining the entire observing community. “A week later they closed for three days. I don’t know whether they paid or not, but we were all left shaken,” recounts an administrator after an extortion-related live broadcast (E5). This finding not only confirms the existence of an emotional climate of fear but also reveals the active process through which it is produced. Extortionist actors use digital platforms as tools of social engineering, deliberately designing and disseminating content with the explicit aim of manufacturing terror at the community scale. Fear thus ceases to be a byproduct of crime and becomes the primary objective of a coercive communication campaign.
2. Naturalization and learning: Fear becomes a form of “situated” and inherited knowledge. The Sankey diagram links normalized fear with inherited fear and the learning of fear, revealing a process of intergenerational and cultural transmission. “It’s scary to go out late or leave the business unattended. Even when you hear a loud noise, you think it might be an attack. That feeling has become normal,” explains one participant (E3). This normalization produces what the Sankey diagram codes as “moral ambiguity”- an ethical gray zone in which distrust becomes generalized: “Before, we used to share information among businesses; now everyone keeps what they know to themselves. People are afraid of getting into trouble for speaking” (E3). Solidarity is sacrificed on the altar of immediate survival, corroding social capital from within.

### 4.2 Hybrid criminal governance: from territorial fees to platform-based extortion

Extortion emerges as the central mechanism of a hybrid form of criminal governance that combines traditional territorial methods with digital tools to regulate the informal economy and contest the state’s monopoly over coercion. This finding provides empirical substance to conceptualizations of organized crime as a form of social ordering.

a The “fee” (cupo): a tributary system of a parallel state.

Periodic payment, or cupo, functions as an illegal yet systematic tax that structures the local economy. Its enforcement is not chaotic; rather, it follows a logic of sustainable extraction grounded in detailed knowledge of the informal economy. As a mototaxi driver explains: “Extortion is the illegal charge imposed by criminals to let you work, using threats” (E11). The “justification of payment,” a code identified in the Sankey diagram, emerges as a pragmatic rationalization in the absence of viable alternatives: “Some choose to pay the fees to avoid more serious problems” (E9). This system generates a perverse form of “protection” that echoes the creation of “fragile assets” described by Neu (2023), whereby vulnerability is first manufactured and then exploited.

b Platformization and the digitalization of coercion.

An innovative finding is the strategic migration of coercive practices into digital environments. This shift does not imply a deterritorialization of extortion; rather, it represents a digitalization of coercion that complements and amplifies physical territorial control. Extortionists operate as ‘entrepreneurs of fear,’ using platforms such as TikTok and WhatsApp to scale their operations. As noted by participants, these digital tools allow for the viral dissemination of threats, where businesses are ‘marked’ publicly to discipline entire communities simultaneously. This digital dimension enables what Arias (2017) describes as a reconfiguration of criminal governance, allowing actors to maintain anonymity while reducing physical exposure. This phenomenon aligns with recent regional evidence indicating that extortion in Latin America has evolved toward cyber-dependent methods, where smartphones and social networks function as essential infrastructures for maintaining territorial grip (Dammert and Sampó, 2025).

c The shadow of the state: perceived corruption and structural distrust. The circuit of criminal governance is sustained by deep institutional distrust. The “state absence” node in the Sankey diagram represents a primary source of flows toward criminal governance and vulnerability. Participants report not only abandonment but also active suspicions of complicity. “Here, anything goes. Some say it’s gangs; others say there are police involved. I don’t know, but it seems like everything is connected,” states one interviewee (E3). This “perceived corruption” (also present in the Sankey diagram) is crucial, as it transforms complaints about inaction into a certainty of collusion, thereby legitimizing what participants perceive as “legalized extortion.” The result is a vicious cycle: distrust inhibits reporting, which consolidates impunity and reinforces the perception that authorities are either ineffective or complicit.

### 4.3 Coping strategies across the spectrum of tactical confinement

Confronted with this apparatus of coercion, economic agents deploy a repertoire of strategies that oscillate along a spectrum ranging from pragmatic negotiation to fragile forms of community resistance, illustrating conditioned agency in contexts of high vulnerability.

a Tactical confinement and self-protection.

The dominant strategy is a form of defensive withdrawal, or “tactical confinement.” This includes physical self-protection (“They install cameras, bars” – E3), digital self-censorship (e). These are not confrontational strategies but rather practices of avoidance and risk minimization—attempts to become “invisible” to extortionary power. Payment of the cupo represents the ultimate form of this pragmatic negotiation.

b The limits and potentials of collective resistance.

Despite the prevailing “culture of silence” (a node in the Sankey diagram), incipient forms of organization are observable. Informal alert networks exist: “I’ve seen that there are WhatsApp groups among neighbors to warn each other if something happens” (E2). In some sectors, emerging forms of mutualism appear: “Some seek neighborhood organization as a form of defense, because that way we look out for each other” (E15). However, the Sankey diagram links community self-protection with conflict among actors, suggesting the fragility of these ties. Mutual distrust, fueled by fear, acts as a constant barrier. As one merchant summarizes: “There is more distrust among merchants now. Before there was more support… but now many prefer to stay on the sidelines out of fear of reprisals” (E9). This finding nuances models of community resilience, showing that social capital, although present, operates under suffocating pressure that severely limits its transformative effectiveness.

### 4.4 The structural vicious circle: informality, violence, and the erosion of the social fabric

Finally, the analysis synthesizes these findings into a model of a vicious circle of structural vulnerability, clearly represented in the interconnections of the Sankey diagram.

The cycle begins not as a mere economic choice, but as a structural consequence of state absence and institutional intermittency (Díaz, 2023). This institutional void functions as the primary driver of vulnerability, forcing a significant portion of the population into economic informality (the central node that both receives and emits multiple flows), understood as a structural condition of precarity. This informality generates territorial stigma and a population that is economically active yet excluded from state protection networks. Upon this unprotected substrate, criminal governance emerges to provide its perverse form of ‘regulation’ (Arias, 2017). This governance is sustained through the strategic manufacture of normalized fear, which in turn produces a culture of silence and institutional distrust, inhibiting reporting and strengthening the impunity of criminal actors. The final outcome is the naturalization of crime and a profound emotional impact, which closes the cycle by deepening distrust and reluctance to engage with the formal system, thereby perpetuating the state’s absence and reafirming informality as the only apparent horizon. “Extortion is destroying the efforts of people who only want to work honestly. Living and working in fear should not be normal,” states one interviewee (E9). This statement encapsulates the core finding: extortion has succeeded in normalizing the abnormal, establishing a social order in which violence becomes the organizing principle of economic and community life, and where the state appears, at best, as a distant spectator and, at worst, as a tacit accomplice in the architecture of coercion (Table 1).

**Table 1:** Synthesis of extortion mechanisms and socio-emotional impacts in San Juan de Lurigancho.

Dimensions of analysis	Operational mechanisms	Impact on economic agents
Affective Regimes	Digital viralization of terror (WhatsApp/TikTok) and performative violence.	Internalization of fear, hypervigilance, and spatial avoidance (self-confinement).
Criminal Governance	“Cupo” as informal taxation and digitalization of coercion (platformization).	Normalization of extortion as a “cost of doing business” and erosion of agency.
State Intermittency	Selective presence, perceived corruption, and institutional abandonment.	Deepening of structural distrust and institutional silence (non-reporting).
Coping Strategies	Pragmatic negotiation, tactical confinement, and incipient alert networks.	Fragile collective efficacy and sacrifice of social capital for immediate survival.

## 5 Discussion

This study examines how extortion operates in contexts of high economic informality, structural vulnerability, and selective state presence. The conceptual density identified in the Tree Map (Figure 1) and the relational flows visualized in the Sankey diagram (Figure 2) provide a robust empirical basis for this analysis. Figure 1 highlights the central role of digital platforms (WhatsApp/TikTok) in the contemporary landscape of coercion and Figure 2 confirms that these practices are not isolated incidents but part of a self-sustaining flow enabled by an institutional vacuum. The findings from San Juan de Lurigancho provide strong empirical support for the theoretical framework developed in the literature on informality, criminal governance, fear, and coercion, while also extending it by revealing the mechanisms through which extortion is emotionally manufactured, digitally scaled, and normalized as a form of governance within everyday economic life.

### 5.1 Informality, structural vulnerability, and the conditions for extortion

The results clearly confirm the theoretical argument that economic informality functions as a structural condition that produces vulnerability, rather than merely reflecting individual economic choices. As suggested by Kamichi (2023) and Çarkoğlu and Eder (2006), informality operates as a self-reinforcing system: low productivity, unstable incomes, and the absence of legal protection push economic actors to adopt survival strategies that increase their exposure to coercion. The accounts of informal merchants and transport workers in San Juan de Lurigancho show that extortion thrives precisely because economic activity unfolds outside formal normative and protective frameworks. Participants repeatedly described extortion not as an exceptional criminal intrusion, but as a predictable risk inherent to informal work itself.

This finding aligns with Neu’s (2023) notion of “fragile assets,” according to which livelihoods are deliberately rendered vulnerable to coercion. Extortion networks exploit informality by transforming the very act of working into a condition that requires authorization through violence. The cupo functions as a parallel tax that mimics the fiscal logic of the state, while extracting value without providing protection or rights, thereby reinforcing the argument that extortion should be understood as a mechanism of governance rather than as a purely criminal act.

### 5.2 Criminal governance and the hybridization of control

The empirical evidence strongly supports theoretical perspectives that conceptualize organized crime as a form of governance rather than as a deviation from it (Albarracín, 2023; Díaz, 2023). In San Juan de Lurigancho, extortion functions as a regulatory system that organizes access to territory, defines acceptable economic behavior, and disciplines non-compliance through credible threats of violence. The results demonstrate that this governance is hybrid in nature, combining territorial control with digital technologies, giving rise to what can be described as a platform-based extension of criminal authority.

The use of TikTok and WhatsApp to publicly mark businesses constitutes a novel empirical contribution to literature. While previous studies have documented extortion as a territorially embedded phenomenon (Fernández, 2022; Miller and Rettberg, 2024), this research shows how digital platforms amplify coercion beyond physical proximity, enabling extortion networks to intensify fear, anonymize perpetrators, and discipline entire communities simultaneously. This process of partial deterritorialization does not replace local control; rather, it intensifies it by expanding the reach of criminal governance while simultaneously reducing operational risks.

The findings in San Juan de Lurigancho regarding the normalization of extortion as ‘just another cost of doing business’ align with the conceptual framework of Sampó (2021), who defines criminal governance as the creation of an alternative social and economic order that coexists with the State through the management of community interactions. This ‘order-making’ process in SJL demonstrates that extortion is not merely a predatory act but a mechanism to build a perverse form of local legitimacy, a phenomenon also identified by Miller and Rettberg (2024) in Medellín, where merchants assume extortion as a structural component of their economic survival. This normalization suggests that in contexts of high informality and weak state presence, criminal actors successfully institutionalize their authority by providing a predictable, albeit violent, regulatory framework for local commerce.

Furthermore, the hybrid nature of this regulation in SJL—where physical threats are amplified by digital platforms—corroborates the regional analysis by Dammert and Sampó (2025) on cyber-dependent criminal methods. This evolution is further explained by the ‘spectacularization of violence’ described by Erazo Patiño et al. (2024), who argue that transnational groups like Tren de Aragua utilize digital media to enlarge the perceived impact of their violence, creating a climate of fear that overpowers state responses. This digital strategy is not isolated; as Leon (2025) observes in the evolution of Mexican cartels, social media and advanced technology (such as drones and encrypted messaging) have transformed criminal organizations into tech-enabled paramilitary actors. These parallels reinforce the thesis that the Lima case is part of a sophisticated regional evolution where extortion acts as a technology of social control mediated by both physical presence and digital infrastructures, effectively manufacturing pervasive emotional regimes that erode the state’s monopoly on force.

### 5.3 The state: absence, selectivity, and perceived collusion

The findings largely align with critical criminological perspectives that emphasize the selective and ambivalent role of the state in marginalized territories (Baratta, 2004). Participants did not merely describe state absence; rather, they articulated perceptions of selective presence characterized by corruption, inefficiency, and potential collusion. This resonates with Ginocchio’s (2022) argument regarding “legalized extortion,” in which institutional practices blur the boundary between regulation and coercion.

The perception that police forces and criminal actors are interconnected reinforces a climate of institutional distrust, which directly inhibits reporting and collective resistance. In this sense, the findings empirically illustrate how penal selectivity and weak protection mechanisms contribute to the consolidation of criminal governance. Rather than acting as a counterweight to extortion, the state appears in participants’ narratives as an unreliable actor—or even as a complicit one—thereby validating Díaz’s (2023) notion that parallel states emerge in contexts of institutional intermittency.

### 5.4 Fear as a technology of social control

One of the most significant contributions of this study lies in demonstrating how fear operates not merely as an emotional response, but as a deliberately designed technology of social control. The findings empirically corroborate theoretical work that conceptualizes fear as an organizing principle of social life in contexts characterized by high levels of criminality (Warburg and Jensen, 2020; Helfgott et al., 2020). However, this study extends the literature by showing how fear is actively produced, disseminated, and learned.

The digital virtualization of threats confirms existing findings on extortion-shaped emotional climates, while also revealing the mechanisms through which fear becomes internalized and normalized. Fear is transmitted both intergenerationally and socially, becoming a form of situated knowledge that structures spatial practices, moral reasoning, and economic decision-making. This process reflects what Bernhard and Cushman (2022) describe as the “dark side of reciprocity,” whereby individuals accept exploitative arrangements in order to avoid greater harm, even while recognizing their injustice.

### 5.5 Coping strategies, limited agency, and the limits of resilience

The strategies identified, payment, evasion, invisibilization, and limited collective action, closely resemble those documented in Colombia and other Latin American contexts (Miller and Rettberg, 2024; Fernández, 2022). However, the findings underscore the deeply constrained nature of agency under conditions of normalized fear. What may appear as “adaptation” is better understood as tactical confinement: a recalibration of everyday life oriented toward survival that minimizes exposure without challenging the underlying power structure.

While the presence of informal networks and WhatsApp-based alert groups supports theories that emphasize the role of social capital in fostering resilience, the results also reveal the fragility of these mechanisms. Fear erodes trust, producing what Vallejo-Robalino et al. (2024) describe as processes of symbolic and material boundary-making. Collective efficacy does exist, but it is constantly undermined by the risk of reprisals, thereby limiting its transformative potential.

### 5.6 Extortion as a reproducer of social vulnerability

The findings confirm the central thesis that extortion is not merely a consequence of vulnerability, but also an active mechanism through which vulnerability is reproduced. Economic informality, fear, criminal governance, and institutional distrust constitute a self-reinforcing cycle that erodes social cohesion and normalizes violence as a condition for economic participation. This dynamic explains why extortion persists despite its economic irrationality for victims and why interventions focused at the individual level are insufficient to contain it.

The results therefore support a structural interpretation of extortion as an integral component of broader systems of inequality, selective governance, and emotional regulation. By normalizing fear and redefining violence as an expected cost of survival, extortion reconfigures moral frameworks, economic practices, and community relations, consolidating an informal order that operates alongside the state and, at times, through it.

## 6 Implications

These findings suggest that policies focused exclusively on law enforcement or situational crime prevention are unlikely to succeed in contexts of high informality. Without addressing structural vulnerability, institutional trust, and the emotional economies of fear, interventions risk reinforcing the very conditions that sustain criminal governance. The study underscores the need for integrated approaches that combine economic formalization, protection for informal workers, institutional accountability, and community-based mechanisms for trust-building.

This research empirically demonstrates how extortion operates as a form of informal governance rooted in structural informality and sustained by fear, digitally mediated coercion, and institutional ambiguity. By foregrounding the lived experience of extortion, the study connects theoretical frameworks on vulnerability and criminal governance with the everyday realities of marginalized urban economies.

## 7 Conclusion

The study’s results confirm that economic informality operates as a structural condition that produces sustained vulnerability and enables the existence and consolidation of parallel orders of control. In this sense, extortion functions as a systematic informal tax that organizes access to work and territory and consolidates enduring relations of dependence, fear, and subordination. It is therefore a mechanism of criminal governance rather than an isolated criminal phenomenon.

The findings provide clear evidence that fear is not merely an emotional reaction to violence, but functions as an intentional technology of social and economic control. Produced, disseminated, and internalized, fear structures everyday practices, economic decision-making, and the moral frameworks of informal actors. The normalization of fear transforms violence into the everyday horizon of economic activity, thereby ensuring the reproduction of extortionary power without the constant resort to physical force.

A central contribution of this research lies in identifying the digital dimension of criminal governance. The use of platforms such as TikTok and WhatsApp reveals a qualitative transformation in the modus operandi of extortion: threats become viral, public, and scalable. These tools function as infrastructures of coercion, enabling a partial deterritorialization of control, an intensification of the climate of fear, and a reduction in operational costs for criminal actors. In this way, contemporary extortion combines territorial control with digital mediation.

The findings highlight that extortion is reinforced by institutional distrust and perceptions of a selective, ineffective, or complicit state presence. This situation inhibits reporting and weakens forms of collective resistance, thereby contributing to the consolidation of parallel criminal orders. The state appears less as a guarantor of protection than as an ambiguous actor, which, from victims’ perspectives, legitimizes criminal governance as a more predictable order.

Finally, although adaptive strategies and incipient forms of community organization are observed, their actual capacity to act freely or bring about change is severely limited. Practices of payment, evasion, and invisibility facilitate survival but do not challenge existing power structures. Social capital holds potential for resilience, yet it is profoundly eroded by fear, distrust, and the risk of reprisals, which ultimately constrains its transformative capacity.

## Data Availability

The datasets are not publicly available due to confidentiality and ethical restrictions but are available from the corresponding author on reasonable request.
